# Chronic Intestinal Obstruction, Report of Case and Operation

**Published:** 1888-12

**Authors:** Wm. Phipps Munn

**Affiliations:** Allegheny, Pa.


### CHRONIC INTESTINAL OBSTRUCTION: REPORT OF CASE AND OPERATION.*

BY WM. PHIPPS MUNN, M. D., ALLEGHENY, PA.

Case: Mrs. E. A., æt. 31, is the mother of five children, the youngest aged eleven months, the oldest twelve years of age. Two years ago she began to be of a constipated habit, but had no special medical attention. Early in 1887 she became pregnant, and in October was delivered at full term by myself. Her confinement was uncomplicated, and her recovery uninterrupted. She resumed her household duties sooner than I thought advisable, on account of not being able to secure proper help. She never became quite as robust as she had been, and complained of constant dragging pain in the back and pelvis, but especially on the left side; the tendency to constipation continually increased; her general health failed in spite of tonic treatment, and her face assumed a cachectic appearance.

In January I was called to see her on account of a more than ordinarily obstinate attack of constipation, the bowels not having moved for seven days, although enemata had been employed every day. During the next three days she took successively calomel, 20 grains in divided doses, castor oil, sulphate of magnesia and sulphate of soda, but without any effect. Then I prescribed an aloin, strychnia, belladonna and ipecac tablet every three hours; by mistake, she took one of these every hour until twenty had been taken, at the same time using hot water enemata twice daily. No effect was produced beyond excessive tympanites. Digital examination of the rectum showed it to be empty; *per vaginam* a sensitive induration was discovered posterior to, and slightly to the left of, the uterus.

Dr. W. S. Foster now saw her in consultation. Placing her in Sim's position, with the hips well elevated, we introduced the

*Read before the Allegheny County Medical Society, September 18, 1888.

long tube of a stomach pump into the rectum until it encountered some obstacle at or near the sigmoid flexure, which was partially passed after a little careful manipulation, and then injected about a quart of hot water into the bowel. She could feel this passing through the descending and transverse colon, but on rising and using the chamber, only the water retained in the rectum was evacuated; the remainder drained away slowly after several hours, but only one or two small lumps of fæcal matter passed. Five times in the next three days I repeated this maneuver, adding each time an ounce of castor oil to the warm water in the syringe, and she finally had a free *fluid* movement, very large in quantity. This was on the fourteenth day, and all this time, although the abdomen was somewhat distended and the pelvic pain continued as before, there was no elevation of temperature or any other sign of peritonitis.

Following this attack she improved much, but it was found necessary to keep the contents of the bowels in a fluid condition by the daily administration of salines; neglect of this for twenty-four hours was invariably followed by a recurrence of the old symptoms and necessitated a return to the long tube of the stomach pump as before. All this time she kept up the daily use of the hot vaginal douche; but, notwithstanding this, the induration in the posterior *cul de sac* appeared to increase; the left ovary could be isolated by careful bi-manual examination, and though somewhat enlarged and very tender, it was very slightly movable. The right ovary seemed to be normal in size, location and mobility, but was abnormally sensitive. Her pelvic pain radiated from the ovaries and was constantly increasing in severity. Early in March an attack of peritonitis threatened, but was averted by the free and persistent use of saline cathartics. Following this, she gained strength for several weeks, and at the end of the month she was able to be out of bed for a few days, but the improvement was only temporary, and the end of April found her once more bedridden. Two attacks of localized peritonitis occurred between this time and the 10th of June, and each time the patient rallied more slowly. Her appetite began to fail, and even the semi-daily administration of salines failed to keep the contents of the bowels soluble. The constant catharsis was also a severe drain on a naturally strong constitution.

The exact nature of the obstruction could not be determined, but it was certainly at or near the sigmoid flexure. The possible conditions were from, *first*, simple stricture or narrowing of the bowel; *second*, a neoplasm, perhaps malignant, involving the bowel at this point; *third*, constriction by fibrinous bands, resulting from peritonitis; *fourth*, a tube or ovary, probably the left, attached to the bowel, as a result of inflammatory action, and forming the obstruction.

Palliative and expectant treatment and persistent use of electricity having utterly failed, and the patient's condition becoming progressively worse, operative interference seemed the only resort with the view of removing the obstruction if possible; and if not removable, to then stitch the bowel to the abdominal wall and establish a fæcal fistula.

Accordingly, on July 13, assisted by Dr. W. S. Foster, I opened the abdomen by a linear central incision five inches in length. The whole length of the bowel, except the transverse colon, which was inaccessible, was carefully examined for constricting bands, but none were found; no stricture could be discovered; there was no new growth. Thus far the examination was negative.

Pressing the abdominal walls well down, I was able to draw the sigmoid flexure into the wound, and found the *left ovary* cystic and about an inch and a half in diameter, dislocated into the *cul de sac* of Douglas, and adherent by its posterior surface to the last movable portion of the intestine. The uterus was enlarged and slightly retroverted. I carefully tore through the adhesions of the ovary to the sigmoid with my finger-nail, ligated the bleeding hemorrhoidal vessels with silk, and approximated the torn edges of the peritoneal coat of the bowel with a continuous horse-hair suture; then ligated the ovarian pedicle and the Fallopian tube with twisted silk, and removed them both. The right ovary, on examination, proved to be cystic also, and was removed. Adherent to the great omentum there was found a colloid body, about a half inch in length, similar to those I have seen in cases of ruptured ovarian cysts with colloid contents. This was ligated and clipped off, but was unfortunately mislaid, and the opportunity for a microscopical examination was lost.

There was almost no hemorrhage during the operation, and what little blood escaped into the peritoneal cavity was rinsed out with previously boiled hydrant water. After sponging this out the wound was closed, and an impervious antiseptic dressing applied. No suppuration occurred, and the stitches were all removed by the fourteenth day. Convalescence was uninterrupted after the second day, although up to that time persistent bilious vomiting occasioned some anxiety. The bowels were fully evacuated on the fourth day by the use of saline purgatives, and after that moved naturally and unaided, except by an occasional aloin strychnia and belladonna tablet every day or every second day. One slight attack of constipation, lasting four days, occurred six weeks after the operation. Two weeks ago she went alone on a visit to relatives in Cleveland and Toledo, and before starting made two shopping tours in the city. She is still in Ohio, and writes to her husband that her strength does not return rapidly, though her appetite and digestion are good. She makes no mention of the condition of the bowels at present.

I report this case on account of the novel cause of the intestinal obstruction. So long as the bowel-contents were perfectly fluid, they passed the point of obstruction easily; but once they became of normal consistency, and accumulated in ever so small an amount above this attached obstacle to the last movable portion of the bowel, and the ovary would be pushed down between the promontory of the sacrum and the slightly retroverted uterus, closing the entrance to the rectum very much after the manner of a ball valve. The prompt relief following the removal of the ovary is a practical reply to any theoretical objection to the operation.

I wish to state distinctly that this was not a premeditated oophorectomy ; had the ovaries been otherwise healthy, I could simply have freed the bowel from them, and perhaps anchored each ovary to its proper broad ligament by a cat-gut suture, but the peritoneal cavity being already opened, and both ovaries discovered in such a degenerated condition as to be functionally useless and a source of danger if allowed to remain, their removal was determined upon with as little hesitation as if they had been foreign bodies.

In reply to an interrogation, Dr. John Ashhurst, Jr., has kindly given me the following statement of similar recorded cases:

PHILADELPHIA, August 17, 1888.

DEAR SIR—Your letter of 16th inst. is received. In the International Encyclopædia of Surgery, vol. vi., p. 74, I have tabulated thirty-seven cases of laparotomy for obstruction of the bowels from tumor, strictures, ulcers, etc., ten of these thirty-seven cases having ended in recovery. I have since added to my list eleven cases, of which five are said to have terminated favorably, so that the figures now stand thus:

| | |
|---|---|
| Recovered | 15 |
| Died | 30 |
| Undetermined | 3 |
| Total | 48. |

Very truly and respectfully,

JOHN ASHHURST, JR.

In Dr. W. Gill Wylie's report of seventy-nine laparotomies during the year 1887, there are two cases of partial intestinal obstruction from "adherent appendages," and one due to chronic pelvic peritonitis. All of these three cases recovered.

The death rate in Dr. Ashhurst's table is higher than one would expect from such an operation performed antiseptically, This I would explain by supposing that many of the tumors and strictures were malignant in nature, or required resection of a portion of the bowel. In my case only the peritoneal coat of the sigmoid was torn, and this was easily brought together by the continuous horse-hair suture, the operation being otherwise uncomplicated.

